# Genetic Diversity Analysis and Core Germplasm Collection Construction of Radish Cultivars Based on Structure Variation Markers

**DOI:** 10.3390/ijms24032554

**Published:** 2023-01-29

**Authors:** Xiaoyao Li, Lei Cui, Lei Zhang, Yan Huang, Shuting Zhang, Weifang Chen, Xiaohui Deng, Zhenbiao Jiao, Wenjie Yang, Zhengming Qiu, Chenghuan Yan

**Affiliations:** 1 Key Laboratory of Ecological Cultivation on Alpine Vegetables (Co-Construction by Ministry and Province), Ministry of Agriculture and Rural Affairs, Institute of Economic Crops, Hubei Academy of Agricultural Sciences, Wuhan 430063, China; 2 Key Laboratory of Horticultural Plant Biology, Ministry of Education, College of Horticulture and Forestry Sciences, Huazhong Agricultural University, Wuhan 430070, China; 3 Hubei Key Laboratory of Vegetable Germplasm Enhancement and Genetic Improvement, Wuhan 430063, China; 4 Jiangsu Key Laboratory of Phylogenomics and Comparative Genomics, School of Life Sciences, Jiangsu Normal University, Xuzhou 221116, China

**Keywords:** radish, genetic diversity, molecular marker, core germplasm collection, structure variation

## Abstract

Radish is an economically important root vegetable worldwide. In this study, the 217 cultivated radish accessions were collected and genotyped. To detect the genotypes of these accessions, a total of 24 structure variation (SV) markers distributed on nine chromosomes were employed to analyze genetic diversity and construct a core germplasm collection of radish. The results of polymorphism information content (PIC) indicated a good polymorphism of these SV markers. Population structure analysis and principal component analysis (PCA) results showed that the 217 radish accessions fell into three main populations (P1, P2, and P3). Genetic diversity analysis showed that these populations were highly associated with geographical distribution. The values of the fixation index (*F_ST_*) indicated a high genetic diversity between P2 and P3, and a moderate genetic diversity between P1 and P2, and P1 and P3. Furthermore, the 43 core germplasm were exploited for creating cytoplasmic male sterility (CMS) lines and cultivating new radish varieties. The high genetic diversity of 217 radish germplasms will not only provide valuable resources for future genetic mapping and functional genomic research, but also facilitate core germplasm utilization and the molecular breeding of radish.

## 1. Introduction

Radish (*Raphanus sativus* L., 2n = 18) is one of the most important vegetables worldwide, especially in Asia. Radishes are cultivated as vegetable crops, oil crops, cover plants, ornamental plants, and medical plants [1]. The cultivated radish is classified into five varieties, including the Asian big radish (*R. sativus* var. *hortensis*), European small radish (*R. sativus* var. *sativus*), oil radish (*R. sativus* var. *chinensis*), black radish (*R. sativus* var. *niger*), and rat-podded radish (*R. sativus* var. *caudatus*) [2]. In addition to the several domesticated cultivars, three wild radishes of *R. raphanistrum*, *R. maritimus*, and *R. landra* have been discovered in the *Raphanus* [3]. *Raphanus* is regarded as a good model for investigating population genetics and gene flow among species and their relatives [4]. Additionally, the hybrid offspring between the cultivated and wild radish are fertile [5].

The analysis of genetic diversity and population structure can reveal the genetic relationships of the investigated populations, and it is essential for large-scale association mapping and genomic selection [6]. Generally, there are three main methods for evaluating genetic diversity and population structure, including morphological analysis, biochemical analysis, and molecular markers [7]. The phenotype of plants tend to be influenced by the environment and breeding history, and, thus, the classification of plant populations merely based on phenotype are likely to be inaccurate [8]. Since only part of the enzymes meets the detection conditions, the application of biochemical analysis in genetic diversity studies is limited [7]. Unaffected by the environments and plant growth stages, molecular markers are usually considered as an ideal tool to evaluate the genetic diversity and population structure of plants [9]. To date, molecular markers have been utilized to explore genetic variation of numerous crops, such as maize (*Zea mays* L.) [10], cucumber (*Cucumis sativus* L.) [11], and broccoli (*Brassica oleracea* var. *italica*) [12]. Additionally, molecular markers are also widely applied for hybrid purity testing and DNA fingerprint profiles.

With the development of biotechnologies, three generations of molecular marker technologies have emerged, including restriction fragment length polymorphism (RFLP), simple sequence repeat (SSR), and single nucleotide polymorphism (SNP), respectively [13]. Currently, the application of RFLP has been rarely reported. SSR markers are widely used worldwide due to their advantages, such as a large number of detection outcomes and co-dominant inheritance [14]. PCR amplification products with SSR markers as primers are usually detected by polyacrylamide gel electrophoresis, which has toxic effects on human health [15]. To date, SNP markers have gradually played a core role in molecular detection due to their high automation based on high-throughput platforms. However, the high-throughput detection of SNPs is expensive, and thus conventional laboratories cannot afford it [16]. To achieve a balance between the convenience and cost for detection, a series of large insertion-deletions (Big InDels, >50 bp) markers, also known as structure variation (SV) markers, have been designed based on large-scale sequencing data, rather than based on two parental samplings [17]. Thus, these SV markers can be distinguished by agarose gel electrophoresis, and they can be used for the detection of radish germplasm.

The objective of this study was to investigate the genetic background information of 217 radish accessions collected from Asia, Europe, and Africa. Using the 24 selected SV markers, DNA fingerprint profiles of these collected radishes were constructed, and their genetic diversity and population structure were analyzed. The relationships between different leaf phenotypes of radish subpopulations and their geographical distribution were further explored. Moreover, we identified the 43 core radish germplasms covering 91.88% of the effective alleles. Our findings lay a foundation for further genetic research and molecular breeding of radish.

## 2. Results

### 2.1. Construction of DNA Fingerprint Profiles by Structure Variation Markers

**Figure 1:**
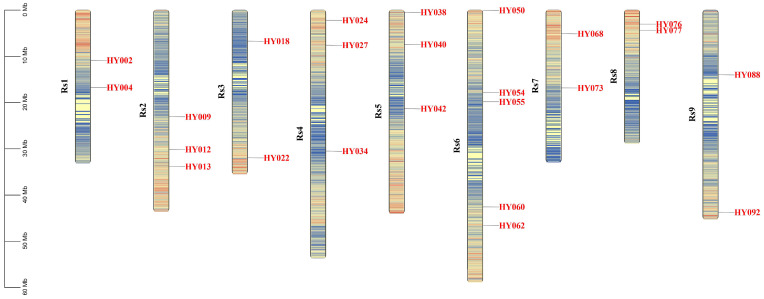
Distribution of 24 SV markers on nine chromosomes.

The 24 SV markers distributed on nine chromosomes were used to identify the genotypes of the 217 collected radish accessions (Figure 1). Differing from simple sequence repeat (SSR) markers, the selected SV markers had only two alleles and a large insertion-deletion variation (>50 bp), which contributed to detection and uniform coding. We obtained 5166 high-quality PCR products from the 5208 PCR reactions and only 0.81% (42/5208) of PCR products were marked by the capital letter ‘X’, which represented low quality and/or missing data. Among PCR products, 4434 single bands (85.14%) were identified as homozygous ‘AA’ or ‘aa’, while 732 double bands (14.06%) were identified as heterozygous ‘Aa’. Based on these bands, we uniformly coded the 217 radish accessions to determine their corresponding molecular identity for subsequent use (Table S2).

### 2.2. Genetic Structure of Radish Accessions

**Figure 2:**
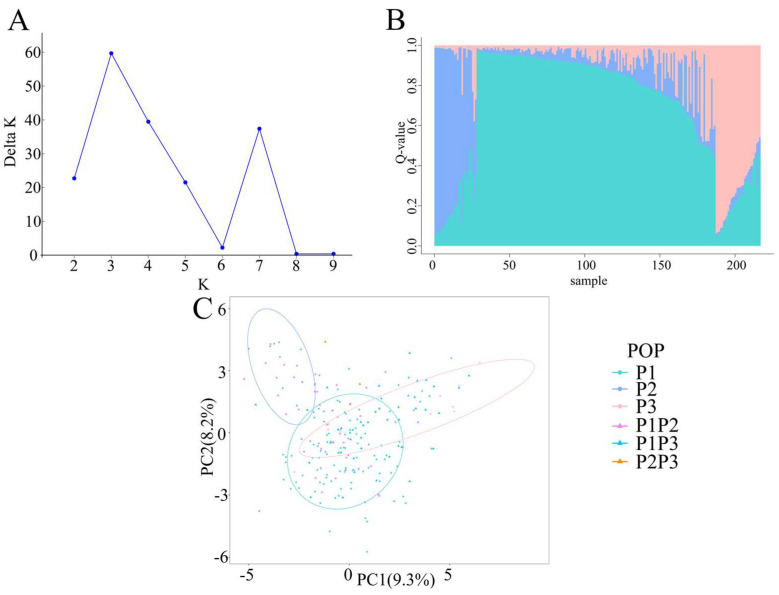
Population structure analysis of 217 radish germplasms. (**A**) ΔK values of different populations. (**B**) Three main subpopulations (P1, P2, and P3) to which 217 radish germplasm resources were assigned. (**C**) Principal component analysis (PCA) of 217 radish accessions. Different subpopulations are exhibited by the different colors: P1, turquoise; P2, cornflower blue; P3, pink; P1P2, purple; P1P3, deep-sky blue; P2P3, orange.

To explore the genetic background of 217 collected germplasms, STRUCTURE 2.3.4 [18] was employed to infer the population structure in this study. The value of ΔK showed a bimodal curve, and the maximum value of ΔK occurred when K = 3 (Figure 2A). The results indicated that all these 217 radish germplasms were assigned to three subpopulations (Figure 2B). Using a membership probability threshold of 0.8, a total of 132 accessions (60.83%) were grouped into one of the three subpopulations. Therefore, 110 accessions, 13 accessions, and 9 accessions were allocated to P1, P2, and P3, respectively. The remaining 85 accessions (39.17%) were considered as admixtures (membership probability < 0.8) [19], including 39 admixtures between P1 and P2 (P1P2), two admixtures between P2 and P3 (P2P3), and 44 admixtures between P1 and P3 (P1P3). The classification information was shown in Table S1. Notably, most of P1 population belonged to Asian big radish (93.63%, 103 accessions). P2 population contained 10 accessions of European small radish (76.92%), which mainly were derived from Europe. In addition, P3 population were all Asian big radish, of which eight accessions (88.89%) were from South Korea. The mixed populations P1P2 and P1P3 had 25 (64.10%) and 38 (86.36%) Asian big radish, respectively. All mixed populations P2P3 (two accessions) belonged to European small radish. In terms of population structure, 217 accessions fell into three categories (P1, P2, and P3) using the hierarchical clustering method based on the phylogenetic tree, which was consistent with the aforementioned three main groups (Figure S2).

To validate the classification of 217 radish accessions, a principal component analysis (PCA) was performed. The results showed that the contribution rates of the first and second principal components were 9.3% and 8.2%, respectively (Figure S3). PCA results showed that 217 radish accessions fell into three subpopulations (Figure 2C), which supported the results of evolution analysis and population structure analysis. The distribution of the remaining admixture populations was relatively scattered. Therefore, the population structure analysis, evolutionary analysis, and PCA jointly illuminated that 217 radish accessions were categorized into three subpopulations (P1, P2, and P3) and their admixtures (P1P2, P2P3, and P1P3).

### 2.3. Genetic Diversity of 217 Radish Accessions

In this study, the observed number of alleles (*Na*) was two (Table 1), which corresponded to the allele number in the primary design of primer pairs of 24 SV markers. The effective number of alleles (*Ne*) varied from 1.2064 to 1.9966, with an average value of 1.7120. The average values of observed heterozygosity (*Ho*) and expected heterozygosity (*He*) were 0.1430 and 0.4018, respectively. The value of Shannon’s information index (*I*) ranged from 0.3128 to 0.6923. Moreover, the polymorphism information content (PIC) ranged from 0.1711 to 0.4991 with an average value of 0.4011. These results indicated that these SV markers had a suitable polymorphism and thus they were qualified for further background selection and evolution analysis.

The analysis of molecular variance (AMOVA) was conducted for further assessing the diversity of radish germplasms. The results showed that the variations among populations and within populations reached 7.36% and 93.64%, respectively (Table 2). The range of pairwise population FST values was 0.063~0.185 (0.122 between P1 and P2, 0.063 between P1 and P3, and 0.185 between P2 and P3), and thus there was a high genetic differentiation between the P2 and P3 populations, and a moderate genetic differentiation between the P1 and P2, and P1 and P3 populations. The gene flow of the three populations was 1.10~3.72 (1.80 between P1 and P2, 3.72 between P1 and P3, and 1.10 between P2 and P3). These results indicate that there was gene flow among populations, which might be due to artificial hybridization by radish breeders.

### 2.4. Geographical Distribution of Radish Germplasms

**Figure 3:**
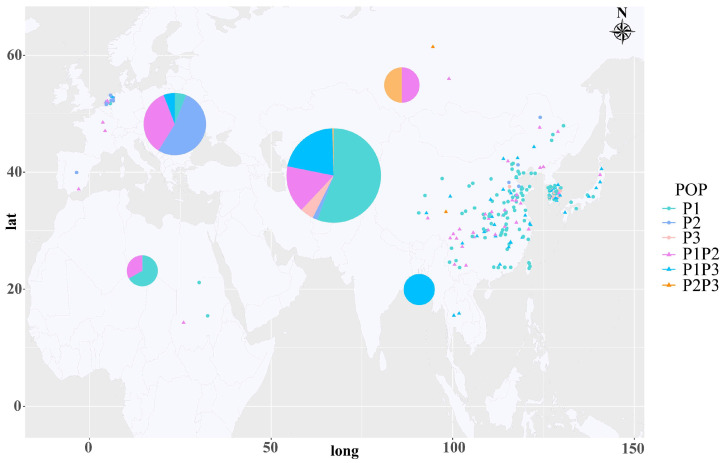
Geographical distribution of collected 217 radish accessions. Different colors represent different populations. P1, turquoise; P2, cornflower blue; P3, pink; P1P2, purple; P1P3, deep-sky blue; P2P3, orange. The regions represent East Asia, Western Europe, North Africa, Southeast Asia, and Russia in pie chart. The color size in each pie chart is proportion to radish accessions number in certain population. The number of the radish accession in each province of China was preliminarily shown in the figure, and the corresponding detailed formation was displayed in Figure S1.

To promote the utilization and breeding of radish, we continuously collected radish germplasms for over twenty years. The classification of subpopulations was highly correlated with the geographical distribution of all radish accessions (Figure 3 and Table S1). P1 consisted of 110 accessions (50.69%), and the majority of P1 population (97.27%, 107 accessions) were collected from Eastern Asia (China, South Korea, and Japan) and southeast Asia regions. P2 represented the type of European small radish, and more than half of P2 (61.54%, eight accessions) came from the Netherlands. Furthermore, almost all of P3 population (89%, eight accessions) was derived from South Korea. In addition, the admixtures were also related to the geographical distribution. Over two thirds of admixture P1P2 was collected from China. The 41 accessions of admixture P1P3 were mainly from Eastern Asia. The two accessions of admixture P2P3 were from Russia and China, respectively. The information of geographical distribution suggested that the admixtures were probably derived from artificial and/or natural hybridization.

### 2.5. Phenotypic Analysis of Subpopulations

**Figure 4:**
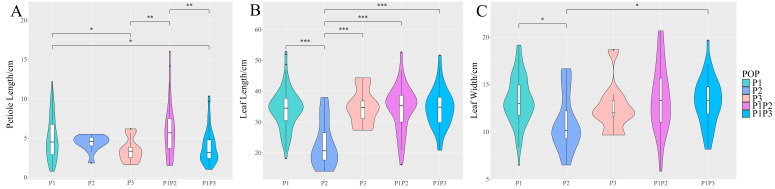
Three leaf-related phenotypes radish subpopulations. (**A**)Petiole length. (**B**) Leaf length. (**C**) Leaf width. * *p* < 0.05; ** *p* < 0.01; and *** *p* < 0.001.

Three leaf-related agronomic traits of 217 collected radish accessions, including petiole length, leaf length, and leaf width, were investigated in this study. Admixture P2P3 was excluded from the analysis since it had only two members. The petiole lengths of P2 and P3 populations concentrated in the range of 1.5–6.5 cm, while other populations showed an obvious dispersed length distribution (Figure 4A). The petiole lengths of P3 population had a significant difference from those of P1 (*p* < 0.05) and P1P2 (*p* < 0.01). Moreover, leaf lengths and leaf widths displayed significant differences between P2 and other populations (Figure 4B,C). The above results suggested that P2 belonged to the type of European small radish with significantly smaller leaf sizes than other populations, and that radish subpopulations were partly associated with three leaf-related traits, reflecting their different origins.

### 2.6. Collection of Core Germplasms

The core accessions were screened on 217 radish accessions using the M-strategy algorithm of Core Hunter II [20,21]. Based on *Na* (Table 3), we determined the optimal screening rate as 20% for constructing the core germplasm collection. Further, we obtained 43 core collections (Table S3), of which 23 accessions (53.5% of the core collections) were from the P1, four accessions (9.3%) were from the P2, and only one accession was from the P3. In addition, the remaining 15 core accessions were from three mixed subpopulations (4 from P1P2, 10 from P1P3, and 1 from P2P3). At the 20% screening rate, these 43 core accessions accounted for over 91.88% of effective alleles (1.4511/1.5793, which was calculated as *Ne* of the core accessions/*Ne* of all the accessions) in the all accessions. These results indicated that the screened 43 core germplasms were able to represent a major part of the genetic information of all accessions.

### 2.7. Utilization of Core Germplasms in Radish

**Figure 5:**
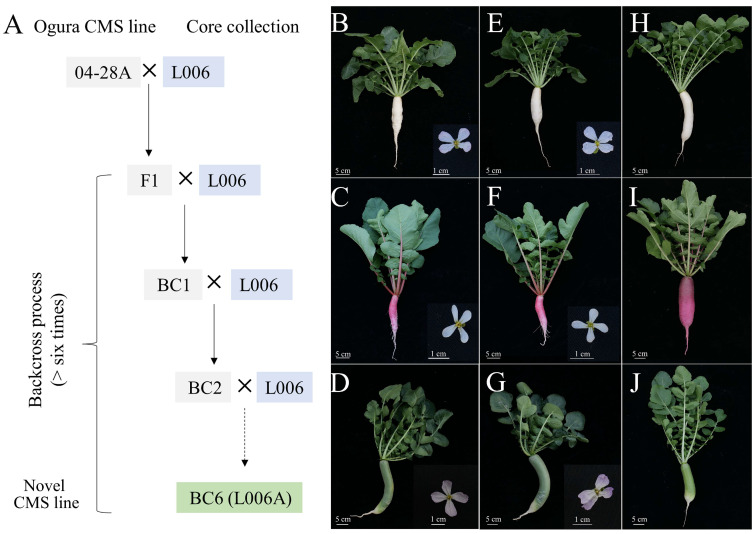
Utilization of three selected core germplasm collections in this study. (**A**) Construction of novel Ogura CMS line L006A. The morphology of the whole plant and its flowers of L006 (**B**), L009 (**C**), L012 (**D**), L006A (**E**), L009 (**F**), and L012 (**G**). The new radish cultivars CR Chuyu No.2 (**H**), ZMR (**I**), and Chucui No.2 (**J**).

To utilize the core germplasms in radish breeding, an Ogura cytoplasmic male sterility (CMS) line 04–28A was employed to construct the novel CMS lines for further hybrid breeding (Figure 5A). Three representative radish accessions, including L006 with white taproot, L009 with red taproot, and L012 with green taproot (Figure 5B–D) from 43 core collections were used as recurrent parents for establishing their CMS lines. Then, we obtained three CMS lines, L006A, L009A, and L012A, after six-generations backcross (Figure 5E–G). Compared with the three core accessions, the stamens of these artificial materials (L006A, L009A, and L012A) lacked normal pollen grains. Subsequently, these CMS lines were applied to breed the corresponding hybrid F1 cultivars, and they were named as CR Chuyu No.2, ZMR, and Chucui No.2, respectively (Figure 5H–J). These core germplasms of radish lay a foundation for our subsequent breeding projects.

## 3. Discussion

### 3.1. Genetic Diversity in Radish Accessions

In this study, population structure analysis, phylogenetic analysis, and PCA results jointly demonstrated that the 217 collected accessions were assigned to three main populations (P1, P2, and P3) (Figure 2). However, based on the morphology and utilization, cultivated radish generally are classified into five varieties, as mentioned above [2]. The classification differences are likely to be attributed to relatively few germplasms of black radish (*R. sativus* var. *niger*) and rat-podded radish (*R. sativus* var. *caudatus*) collected in our study. Our AMOVA results showed that there was a high genetic differentiation between the P2 and P3 and moderate genetic differentiation among main populations (P1 and P2, P1 and P3). In our study, these three populations possessed a strong association with geographical distribution. For example, P1 and P2 were almost derived from Eastern Asia, and Europe, respectively (Figure 3). To our knowledge, these cultivated radishes underwent different domestication events [3] and were domesticated independently [22]. Therefore, the genetic diversity of radish population was strongly associated with the classifications and geographical distributions of their germplasm resources.

### 3.2. Powerful Application of SV Markers

We developed 24 pairs of large fragment insertion/deletion markers (>50 bp), namely SV markers, derived from the whole-genome sequencing data in the study. This was the first report of the assessment on genetic diversity using SV markers in plants. To date, numerous studies have been performed to analyze genetic diversity and population structure in many higher plants, such as rice [23], cabbage [24], maize [10], and cucumber [25]. In these studies, two types of molecular markers, SSR and SNP, have been widely used for constructing fingerprint profiles and selecting the genetic background. Compared to the SSR and SNP markers, SV markers exhibit multiple advantages, such as low-cost, hypotoxicity, and fast detection via agarose gel electrophoresis [26], and relative stability for genetic diversity analysis. In our study, 24 SV selected markers had only two alleles, and thus the genotyping results could be standardized (Table S2). Overall, SV markers are user-friendly, and their results are readable and representative.

In general, the accuracy of the molecular marker detection is improved with the increase in the number of markers. Therefore, the genome-wide SNPs have been gradually exploited for genotyping via the next-generation sequencing (NGS) in higher plants. For example, the 43,735 high-quality genome-wide SNPs were applied to analyze the genetic diversity in maize [10]. In radish, a total of 52,559 SNPs were employed to examine the genetic diversity of 520 accessions using the double-digest restriction site-associated DNA sequencing (ddRAD-Seq) method [3]. However, the accessions can be effectively detected and identified by using a small number of markers. For instance, in one previous study, 97.7% of the 271 pepper varieties were successfully genotyped by using only 27 SNP markers [27]. In another study, 63 inbred cabbage lines were effectively classified by using 20 polymorphic SSR markers [24]. Cucumber DNA fingerprint profiles were constructed using only 16 SSR markers for 382 varieties and 23 SSR markers for 3342 accessions [25,28]. In a recent study, only 10 SNP markers were required for genotyping the 161 broccoli varieties [12]. Thus, it could be concluded that SV markers are desirable tools for genetic background selection and heterozygote detection in laboratories.

### 3.3. Application of Core Germplasms to Breed Improvement

In this study, the Core Hunter II package [20] was applied to construct a radish core collection consisting of 43 core accessions with a good representativeness for our collected radish germplasms. When the screening rate ranged from 10% to 50%, the observed heterozygosity (*Ho*) showed significant differences between the screened germplasms and all of the germplasms (Table 3). Therefore, *Ho* was not suitable to serve as the criterion for screening the core germplasm in this study. Interestingly, as the core germplasm screening rate was 20%, there was no significant difference in the observed number of alleles (*Na*) between the screened germplasms and all the germplasms, indicating that the 20% screening rate could act as a criterion for screening the core germplasm.

Increasing yield and improving commercial quality by enhancing disease and pest resistance are two important breeding goals [29]. For instance, clubroot disease is a widespread severe disease for radish in China [30]. In our previous study, we integrated cytoplasmic male sterility (CMS) and molecular-assisted selection (MAS) technology to culture the novel clubroot resistant cultivar Chuyu NO.1 and identified three loci of clubroot resistance from this novel radish cultivar by genetic analyses [31]. In this study, using three core accessions and the CMS line 04–28A, we cultivated three new CMS lines, L006A, L009A, and L012A, by the traditional breeding method. Furthermore, using these three newly created materials (L006A, L009A, and L012A), three novel hybrids cultivars were generated. To date, more than 50% of radish cultivars have been F1 hybrids. The construction of radish core germplasm collections contributes to the utilization of resources, biodiversity preservation, and anti-disease breed cultivation. Therefore, the radish core germplasm resources remain to be further developed and exploited in future studies.

## 4. Materials and Methods

### 4.1. Plant Materials

A total of 217 radish accessions were collected from Asia, Europe, and Africa over the past twenty years, of which 143 radish accessions (>65%) were collected from China (Figure S1), 40 accessions from South Korea, 13 accessions from Netherlands, 10 accessions from Japan, and 11 accessions from five other countries. The detailed information on 217 radish accessions is listed in Table S1. These radish accessions were germinated and grown in the 72-well plate in the greenhouse of the ‘Yangjiayan’ base of Hubei Academy of Agricultural Sciences (30°32′ N, 114°25′ E, Wuhan, China) on 5 September 2021. Three-week-old seedlings were transplanted into a three-gallon pot. The geographic distribution of the 217 radish accessions was visualized using the RColorBrewer and maptools package (https://CRAN.R-project.org/package=maptools, accessed on 9 May 2022).

### 4.2. Phenotypic Analysis

The phenotypes of six two-month-old individuals from each accession were investigated in our study. At the mature stage of taproot, three leaf-related phenotypes, including petiole length, leaf length, and leaf width, were examined. The experiments were performed with three biological replicates and three technical replicates. Leaf-related phenotypes were plotted using the ggplot2 package [32].

### 4.3. Development of Structure Variation Markers

The young leaf of radish was sampled at 63 days after germination (DAG) for DNA extraction. Genomic DNA was extracted by the CTAB method [33] with a minor modification. Based on the integrated the whole genomic resequencing data of four radish accessions (ENA number: ERP128830) from our previous study and the genomic sequencing data of 520 reported radish accessions [3], 92 SV markers were designed [17]. In this study, 24 SV markers containing only two alleles were used as primers to conduct PCR amplification, and the PCR products were separated by agarose gel electrophoresis. The radish accessions homozygous (AA), homozygous (aa), and heterozygous (Aa) were coded as 0, 1, and 2, respectively. The missing data were filled by the letter ‘X’. The 24 SV markers located on nine chromosomes were visualized using TBtools software [34].

### 4.4. PCR Amplification

PCR amplification was performed by our previously study reported method [35]. The 11 μL PCR system contained 5 μL of 2 × Es Taq MasterMix (CWBIO, Beijing, China), 0.5 μL of forward primer (10 μM), 0.5 μL of reverse primer (10 μM), 4 μL of ddH_2_O, and 1 μL of genomic DNA (50 ng/μL). The procedures of PCR were as follows: pre-denaturation at 95 °C for 5 min, followed by 40 cycles of 95 °C for 15 s, 50–60 °C for 20 s, and 72 °C for 30 s, ending up with a final extension at 72 °C for 5 min. PCR products were detected by 2% agarose gel electrophoresis, and images were captured by the real-time gel imaging system Gel Doc XR+ (Bio-Rad, Shanghai, China).

### 4.5. Principal Component Analysis

A principal component analysis (PCA) of the 217 radish accessions was conducted by FactoMineR [36] and factoextra (https://CRAN.R-project.org/package=factoextra, accessed on 7 May 2022) in R. Firstly, we converted the uniformly coded data into corresponding genotypes. Then, the dimension values were obtained from genotype data. Finally, the two main dimensions were selected for plotting the PCA diagram.

### 4.6. Genetic Structure Analysis

The polymorphism of the 24 SV markers of the collected radish populations was estimated using PopGene software. The original data type (0, 1, 2) was converted to the ‘bp’ type using DataFormater software for further analyses [37]. The missing data were filled by ‘9′. Five core parameters, including the observed number of effective alleles (Na), the effective number of alleles (Ne), the observed heterozygosity (Ho), the expected heterozygosity (He), and Shannon’s information index (I), were estimated using PopGene. In addition, polymorphism information content (PIC) was calculated using PowerMarker software [38].

The population structure was investigated using the STRUCTURE2.3.4 software [18] with the length of the burn-in period of 100,000 and Markov Chain Monte Carlo iterations of 10,000, and the range of K value was set as 1 to 10. The optimal K value was calculated by Structure harvester [39]. A hierarchical clustering analysis of 217 radish accessions was performed based on Euclidean distance. AMOVA analysis was conducted to reveal the variations of the three main populations by GenAIEx [40]. The gene flow levels were calculated according to the formula Nm = 0.25 (1/*F_ST_* − 1) [41].

### 4.7. Screening of Core Germplasm and Their Utilization

Core germplasms of radish with the minimal size were screened using Core Hunter II in R package [20,42]. Five different screening rates (10%, 20%, 30%, 40%, and 50%) were adopted to evaluate the genetic diversities of the germplasms using GenAIEx. By comparing all possible core germplasm sets, we obtained 43 core germplasms and used them to construct sterile lines for further radish breeding.

## 5. Conclusions

In this study, we constructed fingerprint profiles of 217 radish accessions collected during the past twenty years using 24 SV markers. The population structure and PCA results indicate that these 217 accessions fall into three main populations (P1, P2, and P3) and three admixtures (P1P2, P2P3, and P1P3). Genetic diversity analyses revealed a moderate or high genetic differentiation among these populations. In addition, the genetic diversity of our collected accessions was strongly correlated with geographical distribution and leaf-related traits, especially with leaf size. Furthermore, we screened 43 core accessions and further mined their utilization. Overall, we elucidated the genetic background of radish accessions and constructed radish core germplasm collections, based on which we cultivated three new radish varieties.

## Figures and Tables

**Table 1:** Genetic diversity analysis of 217 radish accessions based on 24 SV markers.

Locus	Chromosome	Position	*Na*	*Ne*	*Ho*	*He*	*I*	PIC
HY002	Rs1	10,851,495	2	1.6030	0.1860	0.3770	0.5636	0.3807
HY004	Rs1	16,735,725	2	1.9348	0.2271	0.4843	0.6762	0.4839
HY009	Rs2	23,055,813	2	1.9258	0.2243	0.4819	0.6738	0.4807
HY012	Rs2	30,122,826	2	1.4973	0.1121	0.3329	0.5143	0.3321
HY013	Rs2	33,833,149	2	1.7481	0.2685	0.4289	0.6192	0.4279
HY018	Rs3	6,705,102	2	1.3301	0.0783	0.2488	0.4142	0.2482
HY022	Rs3	31,947,431	2	1.9832	0.0783	0.4969	0.6889	0.4958
HY024	Rs4	2,191,687	2	1.9966	0.1567	0.5003	0.6923	0.4991
HY027	Rs4	7,584,415	2	1.9625	0.0415	0.4916	0.6836	0.4904
HY033	Rs4	30,474,763	2	1.2064	0.0876	0.1715	0.3128	0.1711
HY038	Rs5	454,972	2	1.6381	0.1705	0.3904	0.5782	0.3895
HY040	Rs5	7,436,512	2	1.8864	0.2176	0.4710	0.6627	0.4699
HY042	Rs5	21,313,041	2	1.6647	0.1512	0.4003	0.5888	0.3993
HY050	Rs6	27,231	2	1.8895	0.1349	0.4718	0.6636	0.4708
HY054	Rs6	17,841,141	2	1.9892	0.1982	0.4984	0.6904	0.4973
HY055	Rs6	19,819,455	2	1.2173	0.0876	0.1789	0.3231	0.1785
HY060	Rs6	42,550,591	2	1.6381	0.0968	0.3904	0.5782	0.3895
HY062	Rs6	46,581,963	2	1.9892	0.1982	0.4984	0.6904	0.4973
HY068	Rs7	5,086,840	2	1.4181	0.0461	0.2955	0.4710	0.2948
HY073	Rs7	16,823,631	2	1.9621	0.1852	0.4915	0.6835	0.4904
HY076	Rs8	3,031,541	2	1.3417	0.0599	0.2553	0.4223	0.2547
HY077	Rs8	4,362,064	2	1.9125	0.1256	0.4782	0.6701	0.4771
HY088	Rs9	13,982,718	2	1.6714	0.1792	0.4026	0.5913	0.4017
HY092	Rs9	43,709,005	2	1.6815	0.1204	0.4062	0.5952	0.4053
Mean			2	1.7120	0.1430	0.4018	0.5853	0.4011

*Na*, the observed number of alleles; *Ne*, the effective number of alleles; *Ho*, observed heterozygosity; *He*, expected heterozygosity; *I*, Shannon’s information index; PIC, polymorphism information content.

**Table 2:** Analysis of molecular variance (AMOVA) of 217 radish accessions.

	d.f.	SS	Est. Var.	Percentage of Variation
Among Pops	5	255.901	1.236	7.36%
Within Pops	211	3283.076	15.560	93.64%
Total	216	3538.977	16.795	100%

df, degrees of freedom; SS, sum of squares; Est. Var., estimated variance.

**Table 3:** Genetic diversity parameters of all germplasms and core germplasms.

Screening Ratio	*Na*	*Ne*	*I*	*Ho*	*He*	*uHe*
10%	1.3958 *	1.3112	0.2460	0.0569 **	0.1710	0.2024
20%	1.5833	1.4511	0.3578	0.0682 **	0.2482	0.2781
30%	1.7014	1.5176	0.4159	0.0899 *	0.2866	0.3160
40%	1.7361	1.5289	0.4291	0.0903 *	0.2946	0.3160
50%	1.8125	1.5548	0.4588	0.1056 *	0.3127	0.3341
ALL	1.8750	1.5793	0.4834	0.1438	0.3283	0.3463

*Na*, the observed number of alleles; *Ne*, the effective number of alleles; *Ho*, observed heterozygosity; *He*, expected heterozygosity; *I*, Shannon’s information index; PIC, polymorphism information content. * p < 0.05; **, *p <* 0.01.

## Data Availability

The data presented in this study are available upon request from the corresponding author.

## Supplementary Materials

The following supporting information can be downloaded at: https://www.mdpi.com/article/10.3390/ijms24032554/s1.
