# Supplementary material for: Genetic Diversity Analysis and Core Germplasm Collection Construction of Radish Cultivars Based on Structure Variation Markers
Source: Int J Mol Sci. 2023 Jan 29;24(3):2554. doi: 10.3390/ijms24032554 (PMC9916615; doi:10.3390/ijms24032554)
Supplement: Supplementary file 1 [file ijms-24-02554-s001.zip › Supplemenary Figures.pdf]

Supplementary Figures

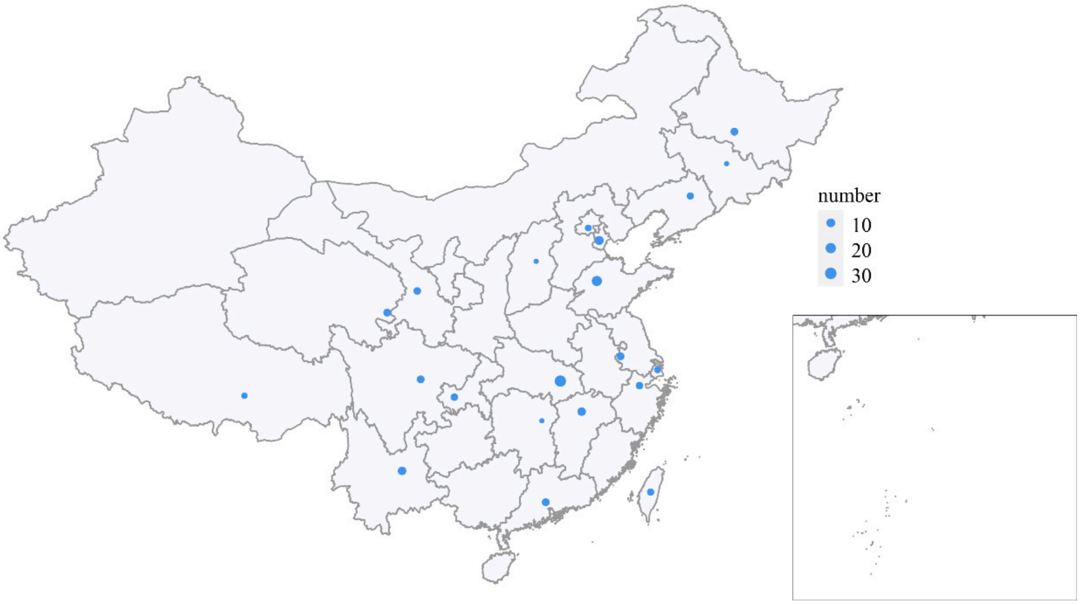

Figure S1 Geographical distribution of the collected radish accessions in China.

Circle sizes represent the number of radish germplasm.

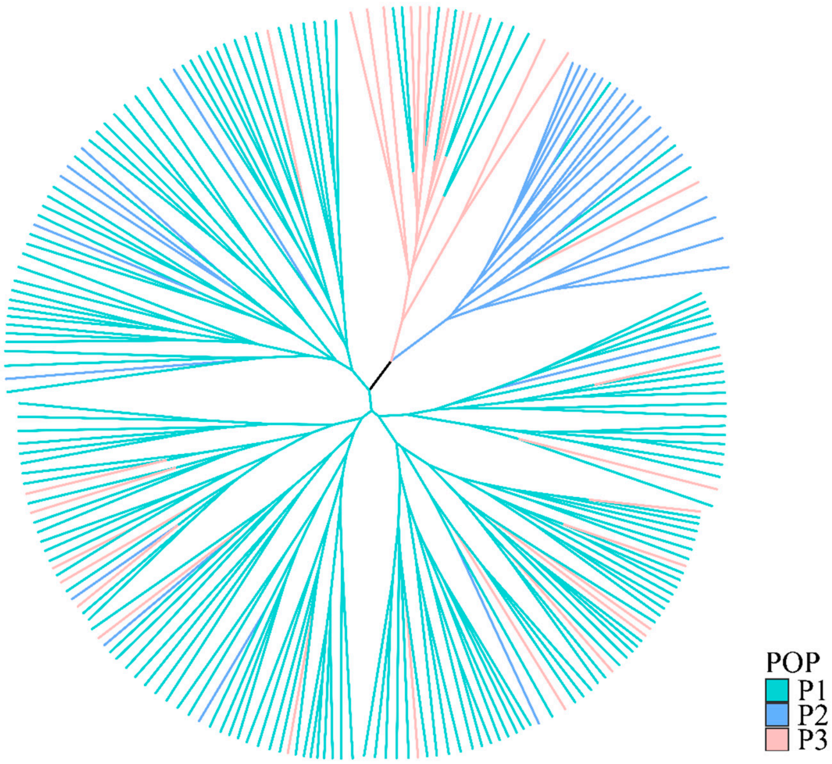

Figure S2 Phylogenetic tree of three main populations of radish using the hierarchical clustering method. P1: turquoise, P2: cornflowerblue, P3: pink.

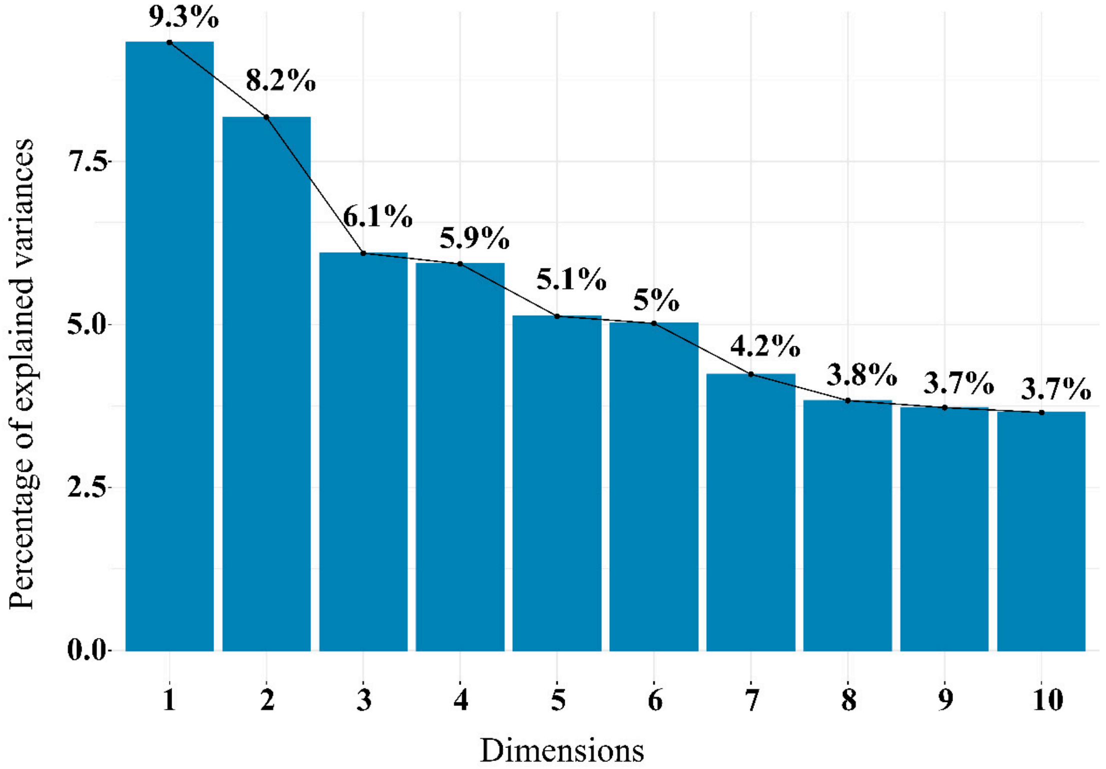

Figure S3 Scree plot of ten principal components for 217 radish accessions.
